# Sexual life in adults treated for brain tumors: a retrospective study

**DOI:** 10.3389/fonc.2024.1483697

**Published:** 2024-10-30

**Authors:** Antonella Leonetti, Guglielmo Puglisi, Marco Rossi, Luca Viganò, Marco Conti Nibali, Lorenzo Gay, Tommaso Sciortino, Luca Fornia, Gabriella Cerri, Lorenzo Bello

**Affiliations:** ^1^ Laboratory of Motor Control, Department of Medical Biotechnologies and Translational Medicine, Università degli Studi di Milano, Milan, Italy; ^2^ Neurosurgical Oncology Unit, Scientific Institut for Research, Hospitalization and Healthcare (IRCCS) Galeazzi Sant’Ambrogio, Milan, Italy; ^3^ Neurosurgical Oncology Unit, Department of Oncology and Hemato-Oncology, Università degli Studi di Milano, Milan, Italy

**Keywords:** sex, sexual satisfaction, desire, brain tumors, quality of life

## Abstract

**Objective:**

Sexual functioning is a multifaceted aspect of human life that can be profoundly affected in patients with glioma. Most frequent symptoms include reduced sexual desire, difficulties in sexual arousal, or low satisfaction. Such symptoms may cause distress or interpersonal difficulties, inevitably resulting in negative outcomes on different domains of patients’ quality of life. Despite this, sexuality is rarely addressed by medical staff and remains understudied. An important question still unanswered is whether sexual dysfunctions in glioma patients correlate with features of the tumor itself, with its treatment, or with the secondary effects of the tumor on the patient’s psychological status. To answer this question, the present study aims to investigate the incidence of sexual life impairments in a very large population of patients with low- and high-grade gliomas, focusing on demographic, clinical, and treatment factors associated with their occurrence and developments.

**Methods:**

A total of 148 patients treated for glioma were evaluated for sexual functioning, i.e., sexual dysfunction (SD), relationship status (RS), intercourse frequency (IF), and sexual satisfaction (SS), by using a specific anonymous questionnaire. Descriptive statistics were utilized to investigate participant characteristics and to evaluate the occurrence of sexual problems. Chi-squared tests were performed to detect the association between “SS” or “IF” and different clinical/demographic factors as well as between “SS” or “IF” and the “subjective–personal skills judgment”.

**Results:**

Results showed no difference between male and female patients, a very low frequency (1.4%) of SD, but a consistent percentage (25%) of subjective deterioration in sexual wellbeing. Notably, 24% of patients reported to have interrupted their relationship after the diagnosis. Chi-squared analyses reveal an association between adjuvant treatments (chemotherapy and radiotherapy) and reduction of IF. Interestingly, “SS” or “IF” was not associated with demographic, clinical, or histomolecular factors.

**Conclusion:**

Our study showed that sexual problems in glioma patients are not uncommon, and they are especially linked to SS, RS, and IF. Specifically, intercourse frequency reduction is associated with the adjuvant treatments. Results highlight the need for improved assessment strategies and interventions tailored to the unique needs of brain tumor patients.

## 1 Introduction

Gliomas are a rare cancer disease (estimate incidence, 25.48 per 100,000) (1), with a significant impact on patient quality of life (QoL). Recent advances in imaging and surgical and adjuvant treatments (chemotherapy and radiotherapy) improve diagnosis, progression, and overall survival. For example, in patients with suspected lower-grade gliomas, the use of a functional neurosurgical approach increases the extent of resection and reduces recurrences, while improving symptoms and reducing cognitive deficit, resulting in a longer and better patient QoL. Currently, most lower-grade glioma patients are expecting to live a long, normal social and professional life (2–4), and therefore, the preservation of QoL has become a crucial issue for surgical and medical neuro-oncologists.

The burden associated with a diagnosis of glioma is dramatic for patients as individuals and for their families. The disease, its diagnosis, and the surgical and adjuvant treatments negatively affect several domains of the patient’s QoL (4), among which the impact on his/her sexual life, which is often underestimated and thus ignored. Cancer patients often face symptoms of sexual life impairment from the time of diagnosis or time of treatment initiation, and these symptoms are likely to persist or even increase in the long term (5). Recent studies reported that, irrespective of the tumor type, 40% of cancer patients, especially young adults, experience sexual problem within the first 2 years following tumor diagnosis (6). The most frequently reported sexual symptoms include reduced sexual desire, difficulty in sexual arousal or orgasm, or low satisfaction with sexual life. Such symptoms may cause marked distress or interpersonal impasse with a substantial negative effect on different aspects of patients’ QoL, often preventing patients from establishing or maintaining an intimate relationship or even building a family. Unfortunately, sexual health is rarely considered or addressed by an oncologist or medical staff (7–10), possibly because of lack of time and/or lack of clarity about relevant factors likely to affect patients’ sexual life in their care path (11). Despite the fact that patients clearly report the need to discuss aspects of their sexual life during hospital consultation (12), uncovering an issue critical for them, the impact of the diagnosis or of its related treatments on sexual life is rarely investigated (13) or mainly limited to the investigation of brain networks potentially involved. The scarce available literature reports an association between right-side resection and difficulty in reaching orgasm and, in men, between temporal lobe resection and reduction of sexual drive and arousal (14). Independent of the specific brain areas involved, sexual symptoms most commonly reported are related to sexual desire (34%) or arousal (37.5%) (10). Such symptoms occur particularly (approximately 50%) in patients affected by LGG and in women (14–16). Limitations of these studies are the small sample size (32–50 patients only), the restriction to lower-grade patients, and the descriptive nature of the analysis.

This study aims at overcoming these limitations and at describing the incidence of sexual life impairments in a large population of glioma patients, including low- and high-grade gliomas, and also investigating the demographic, clinical, and treatment factors associated with their occurrence and developments.

## 2 Materials and methods

### 2.1 Participants and data collection

Participants were selected among patients treated for glioma at our Neurosurgical Oncology Unit.

Patients were included if they fulfilled the following criteria: (I) age ≥ 18 years; (II) absence of severe comprehension deficits affecting the ability to complete the questionnaires; (III) histomolecular diagnosis of LGGs or HGGs; the LGG group included patients with grade II and grade III IDH-mutated and grade II wild-type gliomas, and the HGG group included patients with grade IV wild-type or mutated IDH tumors; (IV) absence of motor deficits; and (V) absence of mood disorders. For each patient, the clinical records relative to tumor (type, grade, and location), medications (anti-epileptic drugs -AEDs- and steroids), and adjuvant therapies (chemotherapy and radiotherapy) were considered for the retrospective analysis, together with socio-demographic characteristics (age and level of education), personal or family history of psychiatric disorder, and current or previous treatments with psychotropic medication.

### 2.2 Study design

Data about sexual life were collected with a self-report questionnaire (see section 2.2.1 for details) administered within the routine neuropsychological/psychological evaluation aimed at assessing the QoL of patients treated at the Oncological Neurosurgery Unit of the IRCCS Ospedale Galeazzi-Sant’Ambrogio for brain tumor resection.

#### 2.2.1 Questionnaire

During the routine neuropsychological/psychological evaluation, patients were assessed with a self-report questionnaire regarding sexual life divided into six sections, specifically designed to investigate the following aspects:

- *Section 1. Demographic information:* sex, sexual orientation, age, partnership status (married/cohabiting, separated/divorced, widowed, or single/never married), and current and past occupational status.
- *Section 2. Medical history and anamnestic information*: cancer diagnosis, other medical diagnosis, drugs taken, and treatment received (number of surgical interventions, adjuvant treatments, and type).
- *Section 3. Organic sexual dysfunctions*: penile erection/vaginal lubrication and ability to reach orgasm.
- *Section 4. Relationship status:* participants were asked about their relationship status before diagnosis and at the time of the questionnaire, and they were asked to indicate the reasons in case of termination of their previous relationships.
- *Section 5. Subjective impact of the disease on sexual pleasure*: the participants were asked to state whether their current sexual experience, i.e., “sexual satisfaction” (interest/desire) and “sexual performances/activities” (frequencies of sexual intercourse), has improved, deteriorated, or remained unchanged compared with the status before diagnosis. If participants reported changes, they were asked to indicate the subjective reasons.
- *Section 6. Subjective judgment about subjects’ following skills*: to communicate personal feelings, to satisfy a partner, to reach orgasm, to be seductive, and to reach a satisfying level of excitement. Each item was scored on a five-point Likert scale (1 = not at all, 2 = a little, 3 = quite a bit, and 4 = very much).

### 2.3 Statistical analyses

Statistical analyses of the anonymized data were performed by using IBM SPSS Statistics Software 20. Descriptive statistics were performed to investigate participant characteristics and to provide occurrence of sexual problems. Chi-squared tests were performed to detect the association between “sexual satisfaction” or “frequencies of sexual intercourse” and various clinical/demographic factors (age, gender, education, IDH mutation, tumor histology, tumor grade, and affected lobe), and between “sexual satisfaction” or “sexual intercourse” and their personal skill judgment.

## 3 Results

### 3.1 Sample

For the statistical analyses, we included all patients who completed the questionnaire, i.e., 148 patients. Of the included patients, 80 (54%) were men and 68 (46%) were women (mean age, 46 ± 12.7 years). Mean interval time from diagnosis was 44.8 months (SD 25.9). Demographic and clinical characteristics of the sample are displayed in **Tables 1**, **2**. According to the histomolecular profile and location, 100 (68%) were diagnosed with an LGG; 73 (58%) patients were operated for a left hemisphere lesion and 120 (81%) patients were also submitted to regular clinical and radiological follow-up in our center. All patients with HGG, included in this study, were submitted to the same adjuvant treatment: radiotherapy fractioned in 30 sessions with temozolomide as the chemotherapeutic agent (average, 10 cycles).

**Table 1:** Demographic characteristics.

**Age, years (SD)**	46 (12.7)
**Educational level, years (SD)**	14.3 (2.8)
Gender	
Female	80 (54%)
Male	68 (46%)
Sexual preferences	
Hetrosexual	145 (98%)
Homosexual	2 (1.4%)
Bisexual	1 (0.6%)
Pt with a relationship before tumor diagnosis	113 (76.4%)
Children	
Yes	88 (59.5%)
No	60 (40.5%)
Employment	
Office worker	47 (32%)
Manager	36 (24.3%)
Laborer	30 (20.3%)
Unemployed	15 (10%)
Retiree	12 (8%)
Student	8 (5.4%)

**Table 2:** Frequency of clinical characteristics.

Tumor grade	
LGG	100 (68%)
HGG	48 (32%)
Hemipshere laterality	
Left	73 (58%)
Right	62 (46%)
Lobe affected	
Frontal	45 (30%)
Temporal	37 (25%)
Parietal	26 (18%)
Insular	25 (17%)
Other	15 (10%)
Stage of care	
Follow-up	120 (81.1%)
Radiotherapy	15 (10.1%)
Chemotherapy	13 (8.8%)
Radiotherapy	
No	93 (63%)
Yes	55 (37%)
Chemotherapy	
No	96 (56%)
Yes	52 (35%)
No. of surgery	
1	87 (59%)
2	47 (32%)
3	9 (6%)
>3	5 (4%)
Antiepileptic drugs	
Yes	100 (68%)
No	48 (32%)
Comorbidity	
Heart disorders	12 (8%)
Urinary incontinence	9 (6%)
Prostate dysfunction	7 (10%)

### 3.2 Descriptive results

Descriptive analyses (percentages) were used to provide the occurrence of organic sexual dysfunction and changes in sexual life, i.e., relationship status and subjective impact of the disease on sexual pleasure, based on questionnaire responses.

#### 3.2.1 Organic sexual dysfunction and relationship status

Only 1.4% of the patients sampled reported “Organic sexual dysfunction”: 1 of 80 men reported sporadic episodes of erectile dysfunction; 1 of 68 women reported vaginal lubrication difficulties. Regarding relationship status, 23 patients (24%) reported to have interrupted their relationship after the diagnosis of a brain tumor.

#### 3.2.2 Subjective impact of the desire on sexual pleasure

Among the 148 patients, 41% (*n* = 62) reported experiencing a sexual change following the diagnosis, with 25% (*n* = 38) reporting a subjective deterioration in sexual wellbeing and 16% (*n* = 25) reporting an improvement. Even though only 25% of patients reported to be dissatisfied with their sexual life, when directly asked about changes in the “frequency of intercourses”, several participants (48%) reported a decrease of frequency after tumor diagnosis, especially after treatments (25% after surgery and 23% after adjuvant therapies). Specifically, the frequency of intercourse was higher before diagnosis in 82% of patients (several times a week in 38% and 2 or 4 times a month in 45%) than after diagnosis (several times a week in 15% and 2/4 times a month in 30%).

Different factors were reported to be subjectively associated with “sexual dissatisfaction” and with a “reduced frequency of intercourse”. Asthenia, due to adjuvant treatments, was associated with both aspects (sexual dissatisfaction and reduced frequency of intercourse) in 47% of patients. Additionally, “relationship issues” emerging during the care path were associated with sexual dissatisfaction in 36% of patients and a reduction in the frequency of intercourse in 41% of patients. “Lack of desire”, followed by “physical problems”, was associated with sexual dissatisfaction in 11% of patients and a reduced frequency of intercourse in 14% of patients. Finally, “drug effects” (AEDs and corticosteroid) were associated with both sexual dissatisfaction and a reduced frequency of intercourse in 4% of patients.

The last part of the questionnaire self-evaluated the patients’ personal feeling relative to their relational and sexual skills before diagnosis; one-third of participants reported a diminished capacity to “satisfy the partner” (32%), or to “reach orgasm” (32%), or “feeling sexual and attractive to others” (27%), or to be “self-confident” (27%) following the diagnosis. Patients ascribed these difficulties to different conditions: cognitive deficits (32%), asthenia (32%), drug effect (22%), alteration of self-image (17%), relationship difficulties (17%), subjective reduction of the motor abilities (15%), effect of adjuvant treatment (chemotherapy and radiotherapy, 15%), mild psychological symptoms (reactive anxiety for the prognosis, 8%), illness-related pain (8%), or loss of interest by the partner (8%).

### 3.3 Factors associated with sexual and relationship changes

Sexual and relationship changes were correlated with several functional and clinical features. The analysis showed that the “frequency of intercourses” was negatively associated with adjuvant treatments (*p* = 0.042), with 55.4% of patients submitted to adjuvant treatments reporting a reduction in the frequency of intercourse, in comparison to patients submitted to the sole follow-up (39.8% of reduction). Interestingly, “sexual satisfaction” or “sexual intercourse” was not associated with any demographic, clinical, or histomolecular factors (see **Table 3**).

**Table 3:** Association between sexual intercourse–sexual satisfaction and demographical and clinical factors.

Factors	Prevalence of diminished SI (%)	*p*-value	Prevalence of diminished SS (%)	*p*-value
**Hemisphere**		0.207		0.382
Left	21		54,2	
Right	27		49	
**Lobe**		0.882		0.674
frontal	63.6		45.5	
parietal	50		43.8	
temporal	60		60	
Fronto-parietal-insular	57.1		71.4	
**Adjuvant treatment**		0.042*		0.384
yes	55.4		55.4	
no	39.8		49.8	
**Sex**		0.770		0.883
Female	52.6		52.6	
Male	58.6		48.3	

In the table, the main explored factors potentially affect SI (sexual intercourse) and SS (sexual satisfaction). The asterisk (*) shows the factor statistically associated with SI. Asteriscs and bold value show the factor statistically associated with SI

## 4 Discussion

In this study, a non-randomized, retrospective analysis was conducted to evaluate sexuality in a large cohort of patients surgically treated for glioma, without previous or concurrent psychiatric disorders. This is one of the largest population-based studies of sexual function ever conducted in the brain cancer population (*n* = 148). The first relevant result is the lower occurrence (1.4%) of organic sexual dysfunctions (i.e., erectile dysfunction/vaginal lubrication impairment) compared with the incidence reported in other studies (14, 16). Despite the negligible percentage of patients with organic sexual dysfunctions, a relevant percentage (25%) of patients reported a subjective deterioration in sexual wellbeing and about half of the sample (48%) reported a decrease of intercourse frequency (two to four times a month). Both changes were significantly associated with treatment (25% after surgery and 23% after adjuvant therapies). These data are also validated by the assessment of subjective perception (subjective factors) related to the worsening of the patients’ quality of sexual life. Adjuvant treatments and related side effects (e.g., asthenia) are reported as determinant factors in 15% and 32% of the sample, respectively. Our results, supporting previous research (16–18), suggest that both the subjective deterioration in sexual wellbeing and the decrease of intercourse frequency experienced by our patients might be due to an indirect effect of the treatments. Treatment side effects on specific aspects of QoL, such as worsening (real or perceived) of physical conditions, lack of social support, or changes in body images mainly due to the radiotherapy, may be responsible. Patients who have undergone adjuvant treatments are at increased risk for developing mood disorders, body image disturbances, and existential concerns, all of which can adversely affect sexual desire, satisfaction, and intimacy. Furthermore, changes in physical appearance, such as hair loss, and decreases in functional independence, such as a temporary inability to drive—common side effects of radiotherapy—may alter self-perception and interpersonal relationships, contributing to sexual distress and relational difficulties. This assumption is also confirmed by the fact that 25% of patients interviewed ended the relationship after diagnosis and that most of the patients (47%) ascribe the reduction of the intercourse frequency to asthenia and relationship issues. Finally, other relevant subjective factors associated with worsening of quality of sexual life are the onset of cognitive symptoms (32%), subjective motor abilities (15%), and psychological symptoms (8%). Although the questionnaire does not allow for an objective assessment of these aspects, this finding is consistent with a recent study (4) showing the importance of functional and psychological impairments in health-related QoL of brain cancer patients. In fact, mood disorders (anxiety or depression) are frequently perceived by patients as the culprits for sexual dysfunctions, particularly in terms of sexual desire (19).

No significant associations were found between the occurrence of sexual dysfunction/satisfaction and demographics (age, gender, educational level, job, etc.), clinical characteristics of the tumor (lobe, hemisphere, and histomolecular profile or the patients’ stage of disease at the time of evaluation), or pharmacological treatments. Notably, this result differs from previous studies reporting tumor location (frontal) or gender (female) as factors linked to sexual dysfunctions (16). Several lines of evidence underline differences between male and female patients in their sexual needs and behavior; i.e., male cancer survivors generally were most concerned about being able to satisfy their partners, while female cancer survivors were most concerned with sex-related changes in their body image. However, gender differences have been found in studies mainly focused on women who have breast or gynecologic cancer and men who have prostate cancer, requiring more invasive and sex-specific surgical and adjuvant treatments than brain tumor.

Although the side effects of oncological treatments seem to be relevant in the emergence of patients’ sexual difficulties, our data suggest that patients’ self-perception may also play a crucial role in their sexual satisfaction. In fact, one-third of the patients perceived themselves as less able to “satisfy the partner” or to “reach orgasm” or “to be attractive” regardless of their physical or clinical conditions.

Taken together, our results highlight that despite the complex multidimensionality of sexual health, in brain cancer patients, some factors clearly emerge as more relevant than others. More than actual organic dysfunctions, which actually play a minor role in the sexual impasse following the diagnosis of brain tumors, the side effects of treatment and the individual perception of sexual inadequacy relevantly impact patients’ sexual life. For this reason, beside the “standard” biological and psychological aspect, an adequate oncologic care of brain cancer patients must consider important, and not underestimate, the side effects of treatments on the quality of sexual life and couple relationships, especially since the patients themselves demand attention to the problem. For this reason, a routine screening for sexual dysfunction, open communication, and a multidisciplinary collaboration to the management of sexual wellbeing in patients with brain cancer is crucial. Interventions may include psychosexual counseling, couple therapy, patient education/empowerment, and also education regarding adaptive sexual practices.

### 4.1 Limitation

In our study, frequency of occurrence estimation was based on self-reports. Sexual health and its vulnerability is an issue prone to stigmatization. We cannot exclude the self-reported data to be biased toward underestimation or to be a subject to “social acceptability” bias. However, precisely because of this issue, sexual problems may be even more disguised by patients in personal interviews, and assessment via self-report may provide more reliable data. Moreover, the present study includes only Italian subjects and manly adults, making it difficult to draw firm conclusions regarding other populations and patients diagnosed before the age of 30.

## Data Availability

The raw data supporting the conclusions of this article will be made available by the authors, without undue reservation.
